# The Impact of Paratuberculosis on Milk Production, Fertility, and Culling in Large Commercial Hungarian Dairy Herds

**DOI:** 10.3389/fvets.2020.565324

**Published:** 2020-10-19

**Authors:** Laszlo Ozsvari, Andrea Harnos, Zsolt Lang, Attila Monostori, Sam Strain, Istvan Fodor

**Affiliations:** ^1^Department of Veterinary Forensics and Economics, University of Veterinary Medicine Budapest, Budapest, Hungary; ^2^Department of Biomathematics and Informatics, University of Veterinary Medicine Budapest, Budapest, Hungary; ^3^Livestock Performance Testing Ltd., Gödöllő, Hungary; ^4^Animal Health and Welfare NI, Dungannon, Northern Ireland

**Keywords:** paratuberculosis, production impact, dairy cattle, milk production, fertility, culling

## Abstract

Paratuberculosis (PTBC) is a chronic disease caused by *Mycobacterium avium* subsp. *paratuberculosis* (MAP), which is common in dairy herds worldwide, although the scale of its impact on herd productivity is unclear. The aim of our study was to determine the differences between MAP ELISA positive vs. negative cows in terms of milk production and quality, reproductive parameters, and culling. The data of five large dairy herds that participated in the voluntary PTBC testing program in Hungary were analyzed. Cows were tested by ELISA (IDEXX Paratuberculosis Screening Ab Test, IDEXX Laboratories, Inc., Westbrook, ME, USA) using milk samples collected during official performance testing. The outcome of the initial screening test involving all milking cows in the herds was used for the classification of the cows. The 305-day milk production, reproduction and culling data of 4,341 dairy cows, and their monthly performance testing results (*n* = 87,818) were analyzed. Multivariate linear and logistic models, and right censored tobit model were used for the statistical analysis. Test-day and 305-day milk production of ELISA positive cows decreased by 4.6 kg [95% CI: 3.5–5.6 kg, *P* < 0.0001 (−13.2%)] and 1,030 kg [95% CI: 708–1,352 kg, *P* < 0.0001 (−9.4%)], compared to their ELISA negative herdmates, respectively. Milk ELISA positive cows had 35.8% higher [95% CI: 17.9–56.4%, *P* < 0.0001] somatic cell count, on average. Test positive cows conceived 23.2 days later [95% CI: 9.2–37.3 days, *P* = 0.0012 (+16.5%)] and their calving interval was 33.8 days longer [95% CI: 13.2–54.4 days, *P* = 0.0013, (+9.7%)], compared to the negative cows, on average. Milk ELISA positive cows were less likely to conceive to first insemination (odds ratio: 0.49, 95% CI: 0.31–0.75, *P* = 0.0013), and required 0.42 more inseminations to conceive [95% CI: 0.07–0.77, *P* = 0.0192 (+13.7%)], on average. Milk ELISA positive cows were culled 160.5 days earlier after testing compared to their ELISA negative herdmates (95% CI: 117.5–203.5 days, *P* < 0.0001). Our results suggest that MAP ELISA positive cows experience decreased milk production, milk quality, fertility, and longevity, which supports the need to control the prevalence of PTBC in dairy herds.

## Introduction

Paratuberculosis (PTBC) is a chronic disease caused by *Mycobacterium avium* subsp. *paratuberculosis* (MAP). Ruminants are most commonly affected, although, the disease has been reported worldwide in many other species including horses, pigs, deer, and rabbits (1).

Herd-level and within-herd true prevalence estimates for dairy herds show a considerable variation among studies, although, the differences in the diagnostic tests, testing strategies, and sampling design make the direct comparison of these estimates difficult (2). The herd-level true prevalence in dairy cattle herds was estimated at 23–34% in Ireland (3), by contrast, it was 75–92% in Denmark (4). Within-herd true prevalence generally ranges from 2.7 to 15% (2). Nielsen and Toft (5) estimated that herd-level true prevalence exceeds 50% and within-herd true prevalence is higher than 3–5% in many countries.

As the disease progresses, PTBC decreases the intestinal absorptive capacity, compromises immunity, and affects productivity of the cows, leading to significant economic losses on the dairy farm depending on the within-herd prevalence (2, 6, 7). The economic loss due to PTBC was estimated to vary from $200 to $1,500 million annually in the US dairy industry alone. However, there is a high level of variability in the published estimates of cow-level losses (2). In France, the economic losses due to clinical and subclinical PTBC were estimated to be 1,940 EUR/case and 461 EUR/case, respectively, in an average French herd of 40 dairy cows with 5,500 kg annual milk production (8). In a 900-cow Hungarian dairy herd, increased mortality and culling rate of MAP ELISA positive cows led to an estimated loss of 166 EUR per cow and a cumulative herd level loss of 238,000 EUR over approximately 4 years (9).

PTBC poses a significant potential risk to human health. Although no causative relationship has been proved between MAP and Crohn's disease so far, the MAP organism is found more frequently in Crohn's patients compared to healthy individuals (10).

Because of the economic relevance of PTBC and its potential risk to human health, it is important to set up effective control programs against MAP. In an international review of PTBC control programs, 46% of the reviewed countries (22 out of 48) had an established control program against MAP (7). The most widely used practices in these control programs were culling the clinical cases (86.4%), hygienic rearing of young animals (77.3%), farm-level biosecurity to prevent introduction of MAP into the herd (77.3%), and testing and culling subclinical cases (72.7%).

Evidence suggests that large herds are more likely to be affected by PTBC (2). The increased risk of PTBC, coupled with the worldwide trend of increasing herd sizes, necessitates the analysis of the impact of infection on large dairy farms. The average size of performance tested dairy herds in Hungary in 2018 was 411 cows (11). Although production loss estimates are available in the literature (12, 13), few studies focus on large dairy herds (14) and typically focus on only a narrow set of production parameters of the herds. However, the overall impact of PTBC on farm productivity might be better assessed, if several parameters are investigated on the farms at the same time.

In Hungary a voluntary PTBC testing program was set up for dairy farms in order to provide help for herd managers to reduce the prevalence and impact of PTBC in their herds. In this program state compensation is available for the dairy farms after taking samples and conducting laboratory PTBC tests up to a maximum amount per individual. In our study we aimed to determine the differences between MAP milk ELISA positive vs. negative cows in terms of milk production and quality, reproductive parameters, and culling on farms participating in the voluntary testing program.

## Materials and Methods

### Data Collection

The inclusion criteria for the farms were as follows: (1) computerized on-farm data recording; (2) continuous participation in milk recording at least since January 1, 2014; (3) herd size of more than 250 cows; (4) implementation of a PTBC screening test for all milking cows in the herd in the voluntary PTBC testing program in February and March 2018; and (5) a willingness to provide data to the authors. Altogether, five large dairy herds were included in the study. The main characteristics of the studied herds are described in Table 1.

**Table 1:** The main characteristics of the studied herds.

**Herd**	**Number of cows^a^**	**305-day milk yield (mean ± SD, kg)**	**Calving interval (mean ± SD, days)**	**Seropositivity (%)**
A	2,011	10,852 ± 2,634	444.5 ± 93.8	4.31
B	441	9,616 ± 1,767	441.5 ± 89.2	3.70
C	905	10,921 ± 1,637	442.5 ± 89.7	4.07
D	593	11,235 ± 2,101	409.5 ± 68.8	10.02
E	1,077	11,242 ± 2,016	442.3 ± 83.1	3.76

a *As of 1 January 2018*.

The surveyed Hungarian herds are located in a warm continental climate (largely Dfb [cold, without dry season, warm summer], partly Dfa [cold, without dry season, hot summer], according to the Köppen-Geiger climate classification). The mean air temperature of the warmest month (July) is usually ≥22°C, whereas that of the coldest month (January) is ≤0°C (15). On all the farms the lactating cows are fed Total Mixed Ration based on maize silage without grazing, and are milked 3 times a day in conventional milking parlors. Dairy heifers and cows are inseminated all year round through artificial insemination. Specific reproductive management practices included the application of a voluntary waiting period of at least 50 days, the use of estrus detection aids, and the application of estrus synchronization. The estrus detection aids used on the surveyed farms were pedometers, activity meters, tail chalking or a combination of these methods. The estrus synchronization protocols generally applied by the herds were OvSynch, CoSynch, and PreSynch-OvSynch. They all used weekly transrectal ultrasonography for pregnancy diagnosis and to determine the stage of the ovarian cycle, as well.

PTBC screening tests were performed on all milking cows (*n* = 4,347) in the studied herds between February 22 and March 22, 2018. Milk samples collected from individual cows during official performance testing were treated with bronopol as a preservative, transported to the laboratory (Livestock Performance Testing Ltd., Gödöllő, Hungary), and examined within 48 h of sample collection. Milk ELISA tests were used for the detection of antibodies against MAP (IDEXX Paratuberculosis Screening Ab Test, IDEXX Laboratories, Inc., Westbrook, ME, USA). Milk samples with S/P ratios ≤0.2 were classified as negative for MAP antibodies according to the manufacturer. If the S/P ratio was >0.2, but <0.3, the sample was classified as inconclusive, whereas samples with an S/P ratio of ≥0.3 were classified as positive for MAP antibodies. The outcome of the initial screening test involving all milking cows in the herds was used for the classification of the cows. Cows with inconclusive ELISA test results (*n* = 6) were excluded from the analyses.

Individual data for all tested cows were collected from the official milk recording database (Livestock Performance Testing Ltd., Gödöllő, Hungary). Two separate datasets were used for the analyses. The first dataset consisted of the monthly performance testing results (*n* = 87,818) of the studied cows between January 2017 and August 2019, which was used for the analysis of test-day milk production and milk composition parameters. The observational unit in this dataset was the test-day record. This dataset contained herd ID, cow ID, date of milk sampling, parity, milk production (kg/day), milk fat content (%), milk protein content (%), somatic cell count (SCC, thousand cells/ml), and MAP milk ELISA test result.

The second dataset was used for the analysis of 305-day milk production, reproductive performance and culling (*n* = 4,341). This dataset included herd ID, cow ID, date of birth, date and result of MAP ELISA testing, parity, the date of the latest calving prior to MAP testing, date and number of the last insemination in the tested lactation, conception to the last insemination in the tested lactation (yes/no), 305-day milk yield (kg), date of the next calving (if applicable), and date of culling (if applicable). The 305-day milk production is calculated by using the formula developed by Wilmink (16) and based on monthly test results. In this dataset, data were available up to July 2019. We analyzed the reproductive performance of the lactation in which the PTBC testing was performed.

### Statistical Analysis

Linear mixed-effects models were applied for the analysis of test-day milk production and milk composition using the nlme package in R (17). SCC data were highly right-skewed, therefore, SCC was log10-transformed, and the model was fitted on the transformed data. Herd, parity (Parity 1, 2, or 3+), and ELISA test result were forced into the models, whereas the calendar month of milk sampling and the two-way interactions were retained in the final models as fixed effects only if significant. The hierarchical structure of the data was taken into account by nesting the lactation number within cow ID in the random effect term of the models. The results of the test positive vs. test negative cows were compared by the emmeans package in R (18), which was also stratified by parity if the interaction of test result and parity was significant. Altogether, the number of samples with missing data on milk fat and milk protein content was 439 (0.50%) and 439 (0.50%), respectively, whereas 427 samples (0.49%) had missing data on SCC.

In the second dataset 3,753 cows with 305-day milk yield data were included. The average DIM at testing was 198.5 ± 133.2 and the median of DIM 182.5 days, and the different DIMs at testing were evenly distributed in the surveyed sample (*n* = 87,818). Therefore, the effect of DIM was not taken into account in the analysis, in harmony with the study of Lombard et al. (19). 3,514, 3,516, and 4,131 cows were included in the analysis of calving to conception interval (CCI), services per conception (SPC), and first-service conception risk, respectively. The analysis of calving interval was restricted to those cows that started their ELISA tested lactation not later than 30 August 2017 to allow a follow-up period of at least 700 days. Of these, there was data on calving interval for 1,408 animals which was included in the analyses. Three hundred five-day milk production, calving to conception interval, calving interval, and the number of services per conception were analyzed by multivariate linear models. First service conception risk was analyzed by multivariate logistic regression. In the analysis of 305-day milk production and reproductive parameters, herd, parity, and ELISA test result were forced into the models, whereas their two-way interactions were retained only if significant. The results of the test positive vs. test negative cows were compared by the emmeans package in R (18).

Times elapsed from testing to culling were compared between ELISA test positive and negative animals using a right censored tobit model (20). The data of 4,287 cows were used for this analysis. The follow up was administratively censored, independently of the life history of the animals, on July 31, 2019, yielding 55.9% censored observations. A three-degree polynomial of time from birth until testing was included in the model as a covariate to capture the non-linear association between age at ELISA testing and survival time from testing to culling. Differences between herds were assessed by including herd as a fixed effect in the model. Culling data were analyzed using the survival package in R (21). Statistical analysis was performed in R version 3.6.1 (22). The level of significance was set to 0.05.

## Results

### Milk Production, SCC, and Milk Composition

The number of performance testing milk samples by ELISA test result and parity is shown by farm in Table 2. Since dry cows were not tested, the total number of tested cows in each herd was less than the cow numbers presented in Table 1. On average, 20.2 test-day records were available for each cow.

**Table 2:** Number of performance testing milk samples and cows by *Mycobacterium avium* subsp. *paratuberculosis* ELISA test result and parity in the studied farms.

	**Test-day milk production and composition**					**305-day milk production, reproduction and culling**				
**Farm**	**No. of milk samples by cow ELISA status**		**No. of milk samples by parity**			**No. of milking cows by ELISA status**		**No. of milking cows by parity**		
	**Negative**	**Positive**	**1**	**2**	**3+**	**Negative**	**Positive**	**1**	**2**	**3+**
A	33,940	1,484	10,867	10,913	13,644	1,619	73	673	417	602
B	7,401	248	3,282	2,614	1,753	364	14	218	84	76
C	15,331	579	6,091	5,751	4,068	755	32	375	224	188
D	9,362	842	2,984	3,526	3,694	449	50	204	131	164
E	18,088	543	6,532	6,800	5,299	948	37	437	292	256
Altogether	84,122	3,696	29,756	29,604	28,458	4,135	206	1,907	1,148	1,286

Milk ELISA test result (*P* < 0.0001), parity (*P* < 0.0001), herd (*P* < 0.0001), month of milk sampling (*P* < 0.0001), the interaction of test result and parity (*P* < 0.0001), the interaction of parity and herd (*P* < 0.0001), and the interaction of test result and month of milk sampling (*P* = 0.0004) were significantly related to test-day milk production. Test-day milk production of the ELISA positive cows was on average 4.6 kg lower (95% confidence interval [CI]: 3.5–5.6 kg, *P* < 0.0001) compared to their ELISA negative herdmates, which corresponded to a relative loss of 13.2%. The difference in milk production between positive and negative cows was larger in higher parities (Parity 2: −6.4 kg/day [−17.6%], Parity 3+: −5.6 kg/day [−15.1%]), although, in primiparous cows, only a tendency for association was found between test result and daily milk production (−1.7 kg/day [−5.7%]; Table 3). Our results show that the daily milk losses observed in ELISA positive cows were significantly greater in lactation 2 (*P* < 0.0001) and lactation 3+ (*P* = 0.0007) compared to lactation 1, but there was no significant difference between lactation 2 and lactation 3+ (*P* = 0.6429).

**Table 3:** Test-day milk production and milk composition (*n*, number of samples) and the 305-day milk yield (*n*, number of cows) by *Mycobacterium avium* subsp. *paratuberculosis* ELISA status estimated from the models.

**Parameter**	***n***	**Diff**.	**95% CI**	**Mean**	**95% CI**	***P***
**Milk yield (kg/day)**						
**Lactation 1**						
Negative	29,023			30.6	30.2–30.9	0.0505
Positive	733	−1.74	−3.48 to 0.00	28.8	27.1–30.5	
**Lactation 2**						
Negative	28,659			36.2	35.9–36.5	<0.0001
Positive	945	−6.37	−7.88 to −4.85	29.8	28.4–31.3	
**Lactation 3+**						
Negative	26,440			36.8	36.4–37.2	<0.0001
Positive	2,018	−5.56	−6.79 to −4.33	31.2	30.0–32.4	
**305-day milk yield (kg)**						
Negative	3,588			11,004	10,924–11,084	<0.0001
Positive	165	−1,030	−1,352 to −708	9,974	9,658–10,290	
**SCC (thousand cells/ml)**						
Negative	83,715			115.7	112.3–119.1	<0.0001
Positive	3,676	+41.4	20.7–65.2	157.0	136.8–180.3	
**Milk fat (%)**						
Negative	83,704			3.67	3.65–3.69	0.0003
Positive	3,675	+0.13	0.06–0.20	3.80	3.73–3.87	
**Milk protein (%)**						
Negative	83,704			3.39	3.38–3.39	<0.0001
Positive	3,675	+0.07	0.04–0.11	3.46	3.43–3.49	

*Diff., difference; 95% CI, 95% confidence interval*.

Herd (*P* < 0.0001), parity (*P* < 0.0001), ELISA test result (*P* < 0.0001), and the interaction of herd with parity (*P* < 0.0001) were significantly related to 305-day milk production. The 305-day milk production of ELISA positive cows was 1,030 kg lower (95% CI: 708–1,352 kg) than that of the negative cows, on average, corresponding to a 9.4% relative loss (Table 3).

SCC was significantly associated with milk ELISA test result (*P* < 0.0001), herd (*P* < 0.0001), parity (*P* < 0.0001), month of milk sampling (*P* < 0.0001), and the interaction of test result and herd (*P* = 0.0082). After transforming the results back to the original scale, we found that test positive cows had 35.8% (95% CI: 17.9–56.4%) higher SCC compared to their test negative herdmates, on average (Table 3). The difference between test positive and test negative cows was more pronounced in Parity 3 and above, although, the interaction of test result and parity was not significant. SCC of the ELISA positive cows was consistently higher in herds A–D, but not in herd E, compared to the ELISA negative cows in the respective herds.

Milk fat content was related to milk ELISA test result (*P* < 0.0001), herd (*P* < 0.0001), parity (*P* = 0.0004), and month of milk sampling (*P* < 0.0001). Test positive cows had 0.13 percentage points (+3.5%) higher milk fat content compared to their test negative counterparts (Table 3). Milk fat content decreased as the parity increased only in test positive cows, although, the interaction of parity and test result was not significant.

Milk protein content was associated with milk ELISA test result (*P* = 0.0033), parity (*P* < 0.0001), herd (*P* < 0.0001), month of milk sampling (*P* < 0.0001), and the interaction of parity and herd (*P* < 0.0001). Test positive cows had 0.07 percentage points (+2.2%) higher milk protein content compared to their test negative counterparts (Table 3). Milk protein content decreased as parity increased regardless of ELISA test status.

### Reproductive Parameters

Herd (*P* < 0.0001), parity (*P* = 0.0016), and ELISA test result (*P* = 0.0012) were significantly related to CCI. Test positive cows conceived 23.2 days later (95% CI: 9.2–37.3 days) than the ELISA negative cows, on average, corresponding to a 16.5% increase in CCI (Table 4).

**Table 4:** Reproductive parameters by *Mycobacterium avium* subsp. *paratuberculosis* ELISA status based on the models.

**Parameter**	***n***	**Diff**.	**95% CI**	**Mean**	**95% CI**	***P***
**Calving to conception interval (days)**						
Negative	3,369			141.3	138.0–144.7	0.0012
Positive	145	+23.2	9.2–37.3	164.6	150.8–178.3	
**Calving interval (days)**						
Negative	1,333			437.1	431.5–442.7	0.0013
Positive	75	+33.8	13.2–54.4	470.9	450.9–490.8	
**Services per conception**						
Negative	3,371			3.05	2.96–3.14	0.0192
Positive	145	+0.42	0.07–0.77	3.47	3.12–3.81	

*n, number of cows; Diff., difference; 95% CI, 95% confidence interval*.

Calving interval was associated with herd (*P* < 0.0001), parity (*P* = 0.0082), and ELISA test result (*P* = 0.0013). Calving interval was 33.8 days (95% CI: 13.2–54.4 days) longer in test positive cows compared to their test negative counterparts, corresponding to a 7.7% increase (Table 4). The difference in the calving interval of ELISA positive vs. negative cows was larger in Parity 1 and 2 compared to the higher parities.

On average, 24.9% of test negative, and 12.9% of test positive cows conceived to first insemination. First service conception rate in the test negative cows was 28.6, 22.1, and 21.2%, whereas in the positive cows it was 12.1, 12.0, and 13.6%, in Parity 1, 2, and 3+, respectively. Herd (*P* < 0.0001), parity (*P* < 0.0001), and ELISA test result (*P* = 0.0005) were significantly related to first service conception risk. Test positive cows were less than half as likely to conceive to first insemination compared to their test negative herdmates (odds ratio: 0.49, 95% CI: 0.31–0.75, *P* = 0.0013).

The number of services per conception was associated with parity (*P* < 0.0001), ELISA test result (*P* = 0.0192), and the interaction of herd with parity (*P* = 0.0363). We did not find a significant relationship between herd and SPC (*P* = 0.1423). Test positive cows required 0.42 (95% CI: 0.07–0.77) more inseminations to conceive, on average (Table 4), which corresponds to a 13.7% increase in SPC.

### Culling

**Figure 1:**
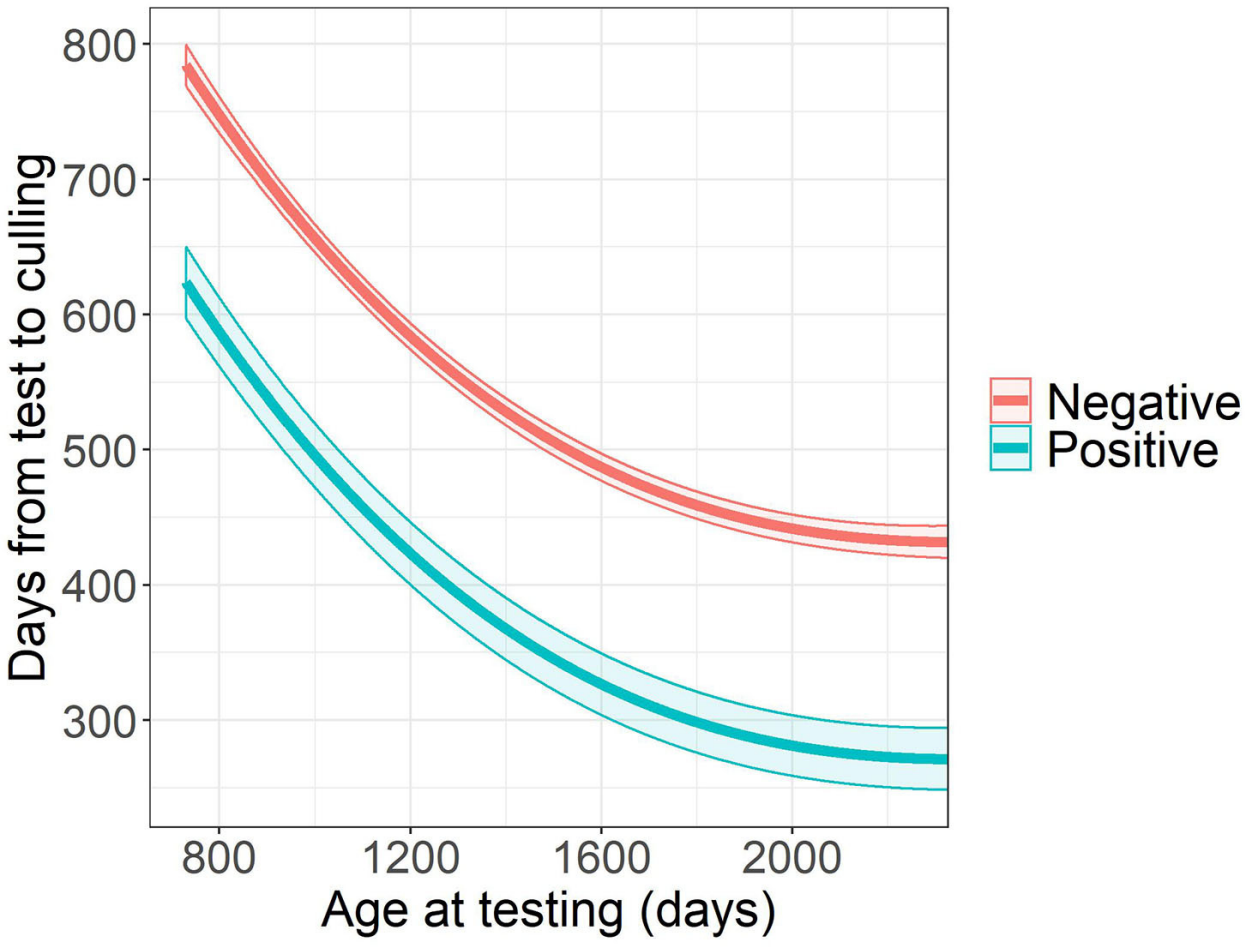
Estimated survival time after testing and its 95% confidence band by *Mycobacterium avium* subsp. *paratuberculosis* ELISA test result^a^. ^a^The graph illustrates the survival time in herd A.

The time from ELISA testing to culling was related to the age at testing (*P* < 0.0001), herd (*P* < 0.0001), and ELISA test result (*P* < 0.0001). Survival time after testing decreased with higher age at the time of ELISA test. Cows with positive ELISA test result were culled 160.5 days earlier after testing compared to their ELISA negative herdmates (95% CI: 117.5–203.5 days, Figure 1).

## Discussion

In general, studies on the impact of PTBC in dairy herds usually investigate only a limited set of production parameters (14, 23). Our study provides novel results about multiple economically important parameters (milk production and quality, reproductive performance, culling) from the same farms, providing a more complete estimate of the losses due to PTBC seropositivity. Furthermore, we focussed on large, intensively managed dairy farms, because of the worldwide trend of increasing average herd sizes and the need for specific estimates for larger herds.

### Milk Production, Composition, and SCC

In our study, milk ELISA positive cows had significantly lower daily milk yield compared to their ELISA negative counterparts, with larger milk losses in higher parities. In the meta-analysis of McAloon et al. (24), combining all case definitions and study designs altogether, the milk production of MAP positive cows diminished by 1.3 kg per day (−4.3%), on average, although there is substantial heterogeneity among studies. We found more pronounced daily milk production losses in ELISA positive cows, which may be explained by a presumably larger proportion of clinical PTBC cases in the studied herds. This is also supported by McAloon et al. (24) and Botaro et al. (25), who found that production losses are much smaller in subclinically affected cows.

Similar to our results, Aly et al. (23) found no significant effect of test status (serum ELISA or fecal culture) on milk yield in the first lactation, but did in subsequent lactations. Tiwari et al. (26) detected an association between MAP and lower milk yield only from the fourth lactation onwards. Martins et al. (27) proposed that serum MAP ELISA positive cows had 0.4–0.6 kg/day higher milk yield in the first lactation, suggesting that increased milk production capacity may be related to susceptibility to MAP infection. In the same study, milk losses amounted to 0.2, 0.8, 1.5, and 2.1 kg/day from the second to fifth lactation, respectively, leading to an accumulated loss of nearly 1,300 kg in the first five lactations in ELISA positive cows, on average.

Production losses in PTBC positive cows are often quantified for the entire lactation. In our study, the 305-day milk production of ELISA positive cows decreased by 1,030 kg (9.4%), on average. In a study of 108 Danish dairy herds using milk ELISA for MAP antibody detection, the loss in 305-day milk production amounted to 540 (8%), 1,057 (13%), and 724 kg (8%), in parity 1, 2, and 3+, respectively (28). Losses in milk production due to PTBC generally amount to 250–1,400 kg per lactation, which is comparable to our results, although, studies vary substantially in the case definition and study design (2, 29).

In the studied herds, significantly higher SCC was observed in ELISA positive cows, with a more pronounced increase from the third lactation onwards. The interaction of test result and herd was significant in the model of SCC, which suggests that herd management influences the increase of SCC in ELISA positive cows. Although the studies show varying results, in general, there is more evidence supporting the theory that PTBC negatively affects udder health (9, 30, 31). Using milk ELISA, Pritchard et al. (13) found elevated SCC in cows with higher risk of being MAP infected, particularly in the second and third lactation. In the same study, mastitis was more likely in high-risk cows compared to their medium- or low-risk herdmates. In a large Portuguese study, MAP serum ELISA positive cows had elevated SCC levels, and the difference between test positive and negative cows increased in higher parities (27). Other studies found no difference in SCC between test positive and negative cows, using fecal culture (14) or serum ELISA (19). One possible explanation is that the progressive weakening of the immune system may cause the increase in SCC in MAP infected cows (27). Our findings also support that the increase in SCC is larger in older ELISA positive cows, which may reflect a more progressed stage of the disease.

Test positive cows had higher milk fat content compared to their test negative counterparts in our study. The results regarding milk fat content are contradictory in the literature. In one study of 58 thousand cows from the UK using milk ELISA, the milk fat production of the cows with high risk of being MAP infected decreased by 34 kg in the first three lactations altogether, compared to the low-risk cows (13). However, milk fat percentage tended to be higher in medium- and high-risk cows in that study, suggesting that the decreased milk fat production was a consequence of the decreased milk yield. Similarly, Jurkovich et al. (30) found significantly higher milk fat percentage in cows shedding compared to those not shedding MAP via feces. However, other studies have failed to find an association between MAP status and milk fat percentage (14, 19). In our study the increase in milk fat content was more than offset by the decrease in milk production, therefore, the amount (kg) of milk fat produced was decreased in the ELISA positive cows. This is in line with the findings of Pritchard et al. (13).

In the present study, ELISA positive cows had higher milk protein content compared to their ELISA negative herdmates. There is no scientific consensus regarding the association of MAP status and milk protein content. Milk protein production of ELISA positive cows decreased by 27.1 kg over the first three lactations, although, milk protein content tended to be higher in high-risk compared to low-risk cows in a large UK study (13). Therefore, decreased milk protein production may be a consequence of lower milk production in ELISA positive cows. In the study of Ózsvári et al. (32), cows with lower daily milk production had higher milk fat and protein percentage, which would partly explain our observations regarding higher milk fat and protein content in the milk of MAP ELISA positive cows. Other studies have found no association or even lower milk protein percentage in the test positive cows (9, 14, 30). Further research is needed to clarify the association of milk protein content and MAP status in dairy cows.

### Reproductive Parameters

In our study, MAP ELISA positivity showed a significant negative association with reproductive performance in dairy cows. Calving interval was more than 1 month longer in test positive compared to test negative cows. Our results agree with a study of seven herds from the US, which found that seropositive cows conceived 28 days later, on average, compared to their seronegative counterparts (33). Similarly, in a study of Iranian Holsteins, cows from MAP PCR positive herds had 30 days longer calving interval compared the cows from PCR negative herds (29). Tiwari et al. (34) reported that the number of open days was 49 days higher in seropositive cows in the first parity, although, they found no association for MAP ELISA status and days open in higher parities. In the study of Jurkovich et al. (30), calving to conception interval and calving interval were longer in cows shedding MAP compared to the non-shedders. Likewise, in a study of six commercial dairy herds, calving interval was increased in cows shedding high levels of MAP compared to low-positive animals (35). However, some studies did not find an association between calving to conception interval, calving interval and MAP status (12, 13, 36).

We found that test positive cows were less than half as likely to conceive to first insemination, and required 0.42 more inseminations to conceive, on average, compared to their test negative herdmates. Very few studies are available on the association of these fertility parameters with MAP status. In the study of Fodor et al. (9), ELISA positive cows required nearly two more inseminations to conceive, on average, compared to ELISA negative cows. However, in that study only a single dairy herd was analyzed. In a large-scale study from the UK, no clear trend between MAP milk ELISA status and the number of services per conception was found (13).

McKenna et al. (31) argued that it is difficult to draw robust conclusions regarding the association of PTBC with fertility, because the differences between MAP positive and negative cows vary by the method of detection. Our analyses were not restricted to the subclinically infected cows, therefore, it is likely that some clinical PTBC cases were also included in our study, leading to larger differences between MAP ELISA positive and negative cows. PTBC weakens the immune system of the cows and impairs the absorptive capacity of the gastrointestinal tract, which might make infected cattle more prone to negative energy balance. In turn these effects may lead to reduced fertility in dairy cows (13, 27, 31).

### Culling

We found a reduced survival time after testing in MAP ELISA test positive dairy cows. In our model the time from ELISA testing to culling was analyzed instead of the age at culling to avoid immortal time bias (37). Controlling for immortal time bias was necessary, because cattle had to remain in the herd for a sufficiently long time period to be tested (they could not be culled earlier, e.g., as heifers prior to testing). Tested cows, by definition, have survived up to the point of being tested. By using the time from ELISA testing in the analysis, we can avoid this “immortal bias” and therefore avoid any upward biased results if “age at culling” was used. Smith et al. (35) found that the detectable infection with MAP was related to increased culling rates, using serum ELISA and fecal culture for MAP detection. Similarly, in a study of nearly 8,000 cows from 38 US dairy herds it was found that cows with positive serum ELISA test results were 1.9-times more likely to have been removed from the herd (19). In a study of a 900-cow Hungarian dairy herd, MAP ELISA positive cows experienced higher culling and mortality rates compared to their seronegative counterparts (9).

Since herd managers were notified about the test results shortly after the laboratory analysis, the increased removal of test positive cows could have been partly caused by the voluntary culling of test positive cows in our study. However, other research shows that the culling rate of MAP ELISA positive cows is higher compared to the negative cows even when the producer is not aware of the test result (19). In addition to the voluntary culling of test positive cows, increased risk of culling may be attributed to impaired milk production and fertility, metabolic disorders due to the malfunction of the intestinal absorption, increased susceptibility to production diseases (e.g., mastitis), and clinical signs of PTBC (6).

Our findings provide novel estimates for the losses occurring in the PTBC seropositive cows in large dairy herds, supporting our expectations that MAP ELISA positivity is linked to a substantial decline in longevity and milk production, especially in higher parities. The extent of production losses found on large dairy farms involved in this study may justify the considerable extra cost of intensive testing to reduce PTBC seropositivity.

## Conclusions

Large dairy farms can experience substantial losses related to PTBC, since cows with positive MAP milk ELISA test had significantly diminished milk production, milk quality, fertility, and longevity. This study supports the need to control PTBC and avoid the significant production losses associated with it.

## Data Availability Statement

The raw data supporting the conclusions of this article will be made available by the authors, without undue reservation.

## Ethics Statement

Ethical review and approval was not required for the animal study because milk samples were collected according to the ICAR standards during the official monthly performance tests. The livestock performance testing was carried out in line with Act LVI of 2019 on the statutory provisions necessary to regulate animal husbandry. Written informed consent was obtained from the owners for the participation of their animals in this study.

## Author Contributions

LO and IF conceived and designed the study. AM collected the data. AH and ZL developed the statistical models and analyzed the data. LO, IF, and SS contributed to conceptualization and writing the paper. LO acquired funding. All authors contributed to manuscript revision, read, and approved the submitted version.

## Conflict of Interest

AM was employed by the company Livestock Performance Testing Ltd. The remaining authors declare that the research was conducted in the absence of any commercial or financial relationships that could be construed as a potential conflict of interest.
